# Trimetazidine-Induced Parkinsonism: A Systematic Review

**DOI:** 10.3389/fneur.2020.00044

**Published:** 2020-02-25

**Authors:** Anna Marielle B. Dy, Lorenzo Luis G. Limjoco, Roland Dominic G. Jamora

**Affiliations:** ^1^Department of Clinical Neurosciences, University of the East Ramon Magsaysay Memorial Medical Center, Inc., Quezon City, Philippines; ^2^Section of Neurology, Department of Internal Medicine, Cardinal Santos Medical Center, San Juan, Philippines; ^3^Department of Surgery, Rizal Medical Center, Pasig, Philippines; ^4^Movement Disorder Service and Section of Neurology, Institute for Neurosciences, St. Luke's Medical Center, Quezon, Philippines; ^5^Department of Neurosciences, College of Medicine – Philippine General Hospital, University of the Philippines Manila, Manila, Philippines

**Keywords:** parkinsonism, trimetazidine, drug-induced, trimetazidine-induced parkinsonism, reversible parkinsonism

## Abstract

**Importance:** Trimetazidine (TMZ) is a medication given to patients with stable coronary artery disease. While it is reportedly well-tolerated, there are increasing numbers of reports of adverse events such as parkinsonism.

**Objectives:** The purpose of this study was to systematically review the currently available literature on TMZ-induced parkinsonism.

**Evidence Review:** A search of Scopus, MEDLINE, EMBASE, the Cochrane Library, the Health Technology Assessment Database, PubMed, Science Direct, and Google Scholar was conducted on or before November 7, 2019. The literature search included cohort studies, prospective and/or retrospective studies, meta-analysis, and other systematic reviews published as an original article, including abstracts and full texts. We included patients taking TMZ who developed one or more of the parkinsonian symptoms of bradykinesia, tremors, rigidity, and postural instability, where these symptoms improved after withdrawal of the said medication.

**Findings:** There are currently five studies on TMZ use and associated parkinsonism. The literature included two case reports, one case series, and one retrospective and one prospective study. We found no results from randomized clinical trials. Overall, 88 patients developed TMZ-induced parkinsonism. Regression of parkinsonism was reported in all of the participants after withdrawal of TMZ. A total of 49 patients (55.7%) had complete regression of symptoms, while 39 patients (44.3%) had significant reduction of symptoms. The duration between TMZ (dose, 60–80 mg/day) intake and onset of symptoms ranged from 4 months to 20 years. The most commonly reported extrapyramidal symptoms were akinesia, rigidity, postural disturbances, and gait disorders, which were usually mild and symmetric.

**Conclusions and Relevance:** The current literature suggests that TMZ can induce parkinsonism that is reversible with drug withdrawal. It is warranted to examine patients, especially the elderly, on TMZ for parkinsonian symptoms and those with pre-existing neurodegenerative diseases. Further studies are needed to assess the risk-benefit ratio of this drug, especially in the elderly age group.

## Introduction

Drug-induced parkinsonism (DIP) is defined as the reversible development of parkinsonian symptoms in patients that are treated with drugs that block the dopaminergic receptor (1–3). It is one of the most important causes of secondary parkinsonism (2). DIP is characterized by its symmetrical presentation and predominantly akinetic-rigid type (1, 3). However, there are instances wherein the clinical manifestation of DIP could not be differentiated from that of Parkinson's disease (PD) or other types of parkinsonism. Thus, some patients are misdiagnosed as having PD and are prescribed with anti-parkinsonian medications that cause no significant resolution of parkinsonian symptoms (4).

DIP is most commonly caused by psychotropic drugs, particularly neuroleptics or the typical antipsychotics such as chlorpromazine, fluphenazine, promethazine, haloperidol, and sulpiride (2). It is the second most common cause of parkinsonism in the elderly next to idiopathic PD (4–6). With the advent of atypical antipsychotics, the incidence of DIP was initially thought to have declined by 30% because their mode of action is more antagonistic on the serotonin receptors than the dopaminergic receptor (2). However, this difference is not apparent among the elderly population, especially when given at high doses (5, 6). Recent studies have revealed that ~50–64% of DIP is caused by drugs other than typical antipsychotics, the most common of which were central dopaminergic antagonists (49%), antidepressants (8%), calcium channel blockers (5%), peripheral dopaminergic antagonists (5%), and H1 antihistamines (5%) (3). The DIP disappeared after withdrawal of the suspected drugs in ~90% of the cases (3). In another study, the incidence of DIP was as high as 33.3%, with trimetazidine (TMZ) and sulpiride being reported as the most frequently used medications (7).

TMZ is marketed as a cytoprotective drug given to patients with stable coronary artery disease and to diabetic and non-diabetic patients with chronic heart failure (8). In the 2016 European Society of Cardiology (ESC) Guidelines on Heart Failure, TMZ was recognized as an effective anti-anginal therapy (Level of Evidence IIb), but it is not recommended in patients with other cardiovascular diseases such as congestive heart failure, acute coronary syndrome, and peripheral arterial disease (9). TMZ is also being used for the treatment of symptoms possibly linked to neurosensory ischemia (such as tinnitus and dizziness) as well as in visual disturbances and age-related macular degeneration (10).

The most commonly reported adverse reactions secondary to TMZ use are primarily gastrointestinal disturbances such as nausea and vomiting (11). Other rare but reversible side effects include thrombocytopenia, agranulocytosis, and liver dysfunction (11). However, there have been increasing numbers of reports that TMZ can cause chorea and parkinsonian symptoms (10, 12–16).

This study aims to review all the available literature on parkinsonism occurring with TMZ use and to systematically investigate if there is a relationship between intake of TMZ and the onset of parkinsonian symptoms and the outcome after withdrawal. Throughout this paper, we will be using the term TMZ-induced parkinsonism to describe parkinsonian symptoms that are completely or at least partially resolved on withdrawal of the said medication.

## Methodology

This systematic review was conducted according to the PRISMA Guidelines (17). The literature search included cohort studies, prospective and/or retrospective studies, meta-analysis, and other systematic reviews published as an original article, including abstracts and full texts. We included patients taking TMZ who developed one or more of the parkinsonian symptoms of bradykinesia, tremors, rigidity, and postural instability, where these improved after withdrawal of the said medication. We searched the electronic databases Scopus, MEDLINE, EMBASE, the Cochrane Library [Cochrane Database of Systematic Reviews, Cochrane Central Register of Controlled Trials (CENTRAL), Cochrane Methodology Register], the Health Technology Assessment Database, PubMed, Science Direct, and Google Scholar. Currently, there are no TMZ trials registered with clinicaltrials.gov. All searches were run on or before November 7, 2019.

The studies were screened by the primary investigator (AMD). Any study that reported on patients developing parkinsonism while on TMZ was included. A second round of screening was performed by the authors based on the abstract and/or full text to identify studies that met the eligibility criteria above. The reference lists of all studies identified during the first round of screening were cross-checked for additional studies that would meet the eligibility criteria. The following data were extracted: first author name, year, study type, sample size number of participants, TMZ dose (if indicated), treatment duration, reported symptoms of parkinsonism, outcome after withdrawal and follow up (if any). There are no limitations made on the study type due to the limited studies available in this field. No assessment of risk of bias was performed since the accepted tools for this type of assessment are designed primarily for randomized controlled trials (RCTs).

Most of the studies (*n* = 4) measured outcomes based on clinical observations (10, 12, 14, 15). One prospective study used the following scales to assess motor and non-motor symptoms: Movement Disorder Society-Unified Parkinson's Disease Rating Scale (MDS-UPDRS), tremor score (TS), postural instability and gait difficulty (PIGD) derived from the UPDRS, 39-item Parkinson's Disease Questionnaire (PDQ-39), Lille Apathy Rating Scale, Non-Motor Symptoms Scale, Montgomery-Asberg Depression Rating Scale, Montreal Cognitive Assessment (MoCA), and Parkinson Anxiety Scale (13). This study also performed cranial magnetic resonance imaging (MRI) and offered a dopamine transporter ligand scan (DaTScan) to all subjects, discussed later on in this review. For the severity of motor symptoms on the basis of MDS-UPDRS scores, patients were classified as mild, moderate, or severe using the cut-off points determined by Martínez-Martín et al. (18).

## Results

The search strategy included applying the following search terms in Google Scholar, Scopus, Medline, the Health Technology Assessment Database, EMBASE, the Cochrane Library, and Science Direct: [trimetazidine AND parkinsonism and human] or [trimetazidine and (Parkinson's disease) AND human] or [extrapyramidal and parkinsonism]; and for PubMed: {(trimetazidine [MeSH Terms]) AND extrapyramidal AND humans [Mesh]} OR {(trimetazidine [MeSH Terms]) AND parkinsonian AND humans [Mesh])} OR {(trimetazidine [MeSH Terms]) AND Parkinson's disease AND humans [Mesh]} OR {(Trimetazidine [MeSH Terms]) AND parkinsonism AND humans [Mesh]}. No limitations were made as to the study type due to the limited studies available in this field. The reference lists of the relevant articles were reviewed to identify eligible studies not captured by these search items.

**Figure 1:**
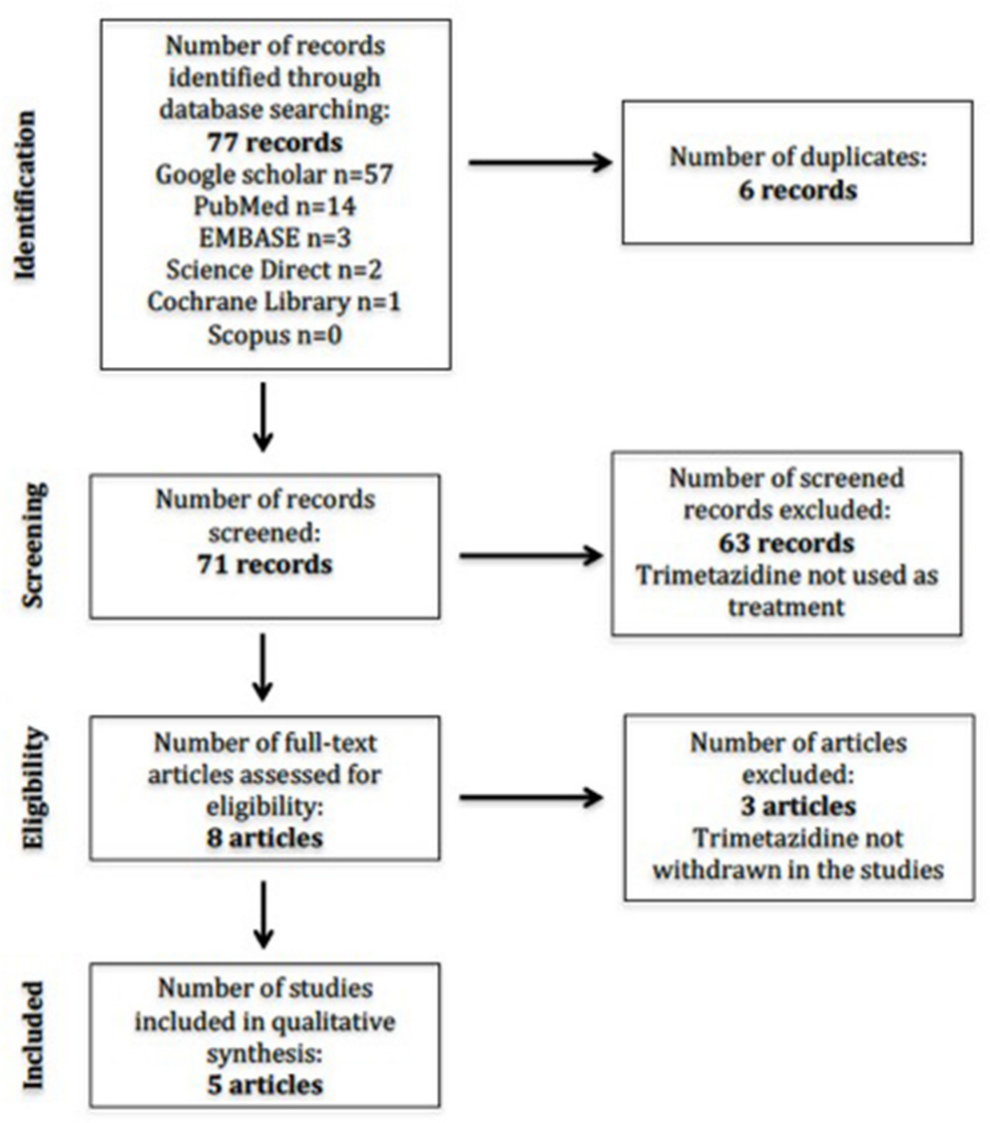
PRISMA flow diagram of information.

The initial search revealed 71 studies after duplicates were removed (Figure 1). Individual database results were Google Scholar (*n* = 57), Scopus (*n* = 0), EMBASE (*n* = 3), the Cochrane Library (*n* = 1), PubMed (*n* = 14), and Science Direct (*n* = 2). No studies were available in the Health Technology Assessment Database. There were eight studies included after initial screening based on title. A second round of screening was done applying eligibility criteria to study abstracts and/or full texts. We translated one study (14) wherein the abstract was in English, but the entire article was in French using Google Translate. Thus, a total of five studies were included for the final full-text review.

### Study Design

There were two case reports, one case series, and one retrospective and one prospective study. The prospective study included 33 patients on TMZ treatment who had previously unrecognized parkinsonian symptoms (13). The intervention carried out was withholding TMZ, and outcomes were measured through neurological and neuropsychological examinations done at baseline, 1 month, and 12 months after TMZ was discontinued (13).

### Patient Characteristics

The total number of participants was 88. In the retrospective study, the total number of patients taking TMZ was 130 (10). However, only 56 of these patients were reported to have neurologic adverse reactions, and only 32 patients taking TMZ were diagnosed with novel parkinsonism or experienced worsening of their pre-existing condition (10). The number of participants per study ranged from 1 to 33. All of the studies were conducted in European countries: Hungary (*n* = 1), France (*n* = 2), Germany (*n* = 1), and Spain (*n* = 1). Table 1 includes the mean age and sex of participants, sample size, indication of TMZ use, dose of TMZ given, treatment duration, reported extrapyramidal symptoms, and follow-up period. One study did not specify the individual doses of TMZ used (10). Another study did not report on the follow up (12).

**Table 1:** Clinical summary of five studies of TMZ-associated parkinsonism.

**References**	**Year**	**Study type**	**Age (mean), in years**	**Gender**	**Sample size (N)**	**Indication for TMZ**	**TMZ dose**	**Treatment duration**	**Extrapyramidal symptoms**	**Outcome after withdrawal**	**Follow up**
Sommet et al. (15)	2004	Case report	91	F	1	Vertigo	70 mg/day	9 months	Facial hypomimia, bilateral bradykinesia, cogwheel rigidity, walking “a petits pas,” postural instability	Favorable, complete regression of symptoms	2 months
Marti Massó et al. (12)	2004	Retrospective	67.6	M = 14 F = 18	32	Vertigo, instability, ocular disease	60 mg/day	11.1 months	Akinesia, gait instability	Favorable; complete regression of symptoms in 20 patients, improvement of symptoms in 12	NI
Sivet et al. (14)	2008	Case report	88	F	1	Visual impairment	70 mg/day	1 month	Choreiform movements, gait disorders, tremor	Favorable, complete regression of symptoms	NI
Masmoudi et al. (10)	2011	Case series	74	M = 14 F = 7	21	Age-related macular degeneration, tinnitus, dizziness, deafness	60–80 mg/day	4 months −20 years	Akathisia, rigidity, tremors, freezing, facial akinesia, micrographia	Favorable; complete regression of symptoms in 16 patients, improvement of symptoms in 5	3–22 months
Pintér et al. (13)	2018	Prospective	70.7 ± 6.6	M =19 F = 14	33	NI	NI	18–120 months	Akinesia, postural instability, rigidity, gait disturbances	Favorable, complete regression of symptoms in 11 patients, improvement of symptoms in 22	12 months

*TMZ, trimetazidine; NI, not indicated*.

### Treatment Outcomes

All 88 patients with TMZ-induced parkinsonism improved after TMZ withdrawal. A total of 49 patients (55.7%) had complete regression of symptoms, while 39 patients (44.3%) had significant reduction of symptoms. The duration between intake of TMZ and onset of symptoms ranged from 1 month to 20 years. The dose ranged from 60 to 80 mg per day.

In one study, 29 (51.7%) patients were taking other drugs capable of inducing parkinsonism or worsening gait stability (sulpiride, cinnarazine, thiethylperazine, flunarizine, fluoxetine, flupentixol, melitracen, diazepam, and omeprazole) and these drugs were withdrawn simultaneously (10).

Three studies reported unmasking or worsening of subclinical parkinsonism after being given TMZ, with improvement in symptoms when TMZ was withdrawn (10, 12, 15). In one study, five patients had persistent symptoms, which included buccolinguofacial dyskinesias, postural tremors, and slight parkinsonism, and an initial diagnosis of idiopathic PD was made. These patients improved slightly on levodopa/carbidopa therapy (10).

To further differentiate TMZ-induced parkinsonism and idiopathic PD, one prospective study used clinical rating scales to assess both motor and non-motor parkinsonian symptoms at baseline, 1 month, and 12 months after discontinuation of TMZ (13). Of the 33 patients with previously unrecognized parkinsonian symptoms on TMZ, 11 had complete resolution of parkinsonian symptoms after TMZ withdrawal. TMZ-induced parkinsonism was found to be mainly characterized by akinesia, rigidity, and PIGD (PIGD scores: 5.3 ± 3.8 vs. 2.0 ± 1.6 points, *p* = 0.006) rather than tremors (tremor scores: 1.5 ± 2.2 vs. 7.7 ± 4.6 points, *p* < 0.001). In addition, TMZ-induced parkinsonism was also more symmetrical (asymmetry index: 3.1 ± 3.6 vs. 40.1 ± 22.2, *p* < 0.001) and milder in severity (MDS-UPDRS Part III scores: 10.5 ± 19. vs. 30.5 ± 11.3, *p* = 0.040) than non-reversible parkinsonism. Although the motor symptoms of TMZ-induced parkinsonism were considered to be generally mild and the MDS-UPDRS scores were lower, patients had worse health-related quality of life as measured by the PDQ-39. Non-motor symptoms were found to be less helpful for differentiating TMZ-induced parkinsonism from idiopathic PD (13).

## Discussion

One of the first cases reported of drug-induced parkinsonism due to TMZ treatment was a case report on an elderly female with vertigo who was on TMZ for 9 months and developed parkinsonian symptoms that completely resolved 2 months after withdrawal of the said medication (15). This was followed by a retrospective study showing that TMZ can cause parkinsonism or worsening of previous extrapyramidal symptoms in 43% of the patients taking TMZ, the clinical characteristics of which were similar to those of other drug-induced parkinsonism but with a greater tendency to induce poor balance and postural instability (12). Since then, there have been increasing numbers of reports on this adverse effect. In a recent large, cross-sectional study, the records of patients aged 40 years or more diagnosed with angina and subsequently taking TMZ were studied (19). Of the 2,169 patients taking TMZ included in the study, 2.9% had newly diagnosed parkinsonism (19). However, these were not included in this review since reversibility of the symptoms was not reported.

There are currently five reported studies on parkinsonism and TMZ use. These studies have shown that TMZ can induce secondary and reversible parkinsonism and that it can unmask subclinical neurodegenerative parkinsonism. Table 1 summarizes the findings in this study.

The most commonly reported extrapyramidal symptoms were akinesia, rigidity, postural disturbances, and gait disorders (10, 12–15). These features were usually mild and symmetric (13). However, there have also been reports on non-motor features of TMZ-induced parkinsonism such as depression, anxiety, and apathy (12, 13). A favorable outcome was seen in all of the studies after withdrawal of TMZ, which is the main characteristic feature of drug-induced parkinsonism.

Cognitive impairment was also implicated in patients with drug-induced parkinsonism in some studies. The outcome measured through average MoCA score showed that the TMZ-associated parkinsonism group has lower values compared to the general PD population (23.6 ± 3.6) (13). However, the withdrawal of TMZ did not show any improvement in cognition, so its impairment may not be induced by TMZ treatment (13).

The adverse effects were predominantly seen in the elderly age group rather than in younger adults. The age of patients with TMZ-induced parkinsonism ranged from 65 to 81 years old. In a prospective study, the age was comparable to those patients with irreversible parkinsonism (68.4 ± 4.8 vs. 71.8 ± 7.2; *p* = 0.248) (13). This emphasizes the importance of age as a cofactor in this type of parkinsonism, which could be due to the gradual depletion of dopamine levels and poorer renal function in the elderly and multiple drug interactions (10, 12, 13).

In patients with subclinical neurodegenerative parkinsonism, TMZ has been shown to unmask or worsen these symptoms. However, in contrast to patients with TMZ-associated parkinsonism, the main symptom observed was asymmetric resting tremors that showed some improvement after TMZ discontinuation, with further improvement only achieved with anti-parkinsonian therapy (13).

Some studies made use of DaTScan, showing abnormal findings in patients with degenerative parkinsonism but normal results in patients with drug-induced parkinsonism (5, 13). DATs are presynaptic proteins on the membrane terminal of dopaminergic neurons that control dopaminergic neurotransmission. The degree of uptake can be detected by single-photon-emission computed tomography and positron-emission tomography scans using DAT ligands such as DaTScan. DaT uptake in the striatum is reduced in patients with idiopathic PD, even during the early stages of the disease, while it has negligible or normal values in patients with DIP (4, 20).

TMZ is a dibasic compound, coming from a piperazine derivative with the following chemical structural formula: 1-[2,3,4-trimethoxibenzyl]-piperazine (10). It is primarily being marketed as a cellular anti-ischemic agent but is also being prescribed to patients with neurosensory ischemia (such as tinnitus and dizziness), visual disturbances, and age-related macular degeneration (8–10).

The exact mechanism of action by which TMZ induces or worsens parkinsonism is not yet fully understood, but there are studies that have shown that the piperazine core in TMZ is the same structure as that found in cinnarizine and flunarizine, which are being marketed for dizziness and for migraine or vascular headaches, respectively. These drugs belong to the calcium channel blocker group and have also been found to induce movement disorders via blockade of striatal D2 dopamine receptors in the basal ganglia (5, 10, 20). The blockage of D2 receptors in the striatum leads to the disinhibition of striatal neurons at the origin of the indirect pathway, followed by disinhibition of the subthalamic nucleus, which causes hypokinetic movement disorders such as parkinsonism. Meanwhile, other drug-induced movement disorders such as akathisia and chorea are due to the dopamingergic hypersensitivity resulting from long-term D2 receptor blocking (4). This explains why akathisia and choreiform movements disappeared after drug withdrawal (10, 12).

Several limitations should be considered in the interpretation of the findings of our study. This review included all study designs (case report, case series, and retrospective and prospective studies). Assessment of risk of bias across studies was not done due to the varying study designs used and the wide range of sample size. One article was not in English and was translated in order to be included in the study to increase the sample size (14).

## Conclusion

The current literature suggests that TMZ can induce parkinsonism that is reversible with drug withdrawal. Despite the increasing number of reports of TMZ-induced parkinsonism, this risk remains poorly recognized by clinicians. Hence, it is highly recommended to examine patients, especially the elderly, on TMZ for parkinsonian symptoms as well as those with pre-existing neurodegenerative diseases. Further studies are needed to assess the risk-benefit ratio of this drug, particularly in the elderly population.

## Data Availability Statement

All datasets generated for this study are included in the article/supplementary material.

## Author Contributions

AD: acquisition of data, statistical analysis and interpretation, and writing of the initial draft. LL: acquisition of data, analysis and interpretation, and critical revision of the manuscript for intellectual content. RJ: study concept and design, analysis and interpretation, critical revision of the manuscript for intellectual content, and study supervision.

### Conflict of Interest

The authors declare that the research was conducted in the absence of any commercial or financial relationships that could be construed as a potential conflict of interest.
